# 

**Published:** 1884-05

**Authors:** 


					﻿Whole Number,
UCCCLXXXII.
May, 1884.
Number 5,
Vol. XLVli 7^
TITZE
CHICAGO
MEDICAL
OURNAL & EXAMINER
CHICAGO:
PUBLISHED BY THE CHICAGO MEDICAL PRESS ASSOCIATION
188 Clark Street.
tSFRaad the Notice of this Journal among Advertisements
				

